# Attitudes regarding endoscopic third ventriculostomy and choroid plexus coagulation (ETV+CPC) and the effect of training at CURE Children's Hospital, Uganda among North American pediatric neurosurgeons

**DOI:** 10.1186/2045-8118-12-S1-O10

**Published:** 2015-09-18

**Authors:** Jay Riva-Cambrin, Heather Spader, Abhaya Kulkarni, Benjamin Warf, John Mugamba, Peter Ssenyonga

**Affiliations:** 1 University of Utah, USA; 2 University of Toronto, Canada; 3 Harvard University, USA; 4 CURE Hospital, Uganda

## Introduction

The ETV+CPC technique has permeated from CURE Hospital in Uganda to North America and neurosurgeons have travelled to Uganda to learn the nuisances of this technique. The goal of this study was to determine current attitudes of North American pediatric neurosurgeons towards ETV+CPC and the effects of training at CURE on attitude and clinical practice.

## Methods

The electronic cross-sectional survey comprised of general questions and specific cases examining practices and attitudes regarding shunts, ETV, and ETV+CPC for the management of infant hydrocephalus. Twelve surgeons were identified as having trained at CURE and each surgeon was matched with 4 other pediatric neurosurgeons based on length of practice. The CURE-trained neurosurgeons were asked about their ETV+CPC attitudes and management before and after training.

## Results

Overall response rate was 67% (37/55). Of all respondents, 41% thought ETV+CPC was superior to CSF shunting, 24% thought it was inferior, and 8% felt they were equivalent. Of the CURE trainees, 38% did not offer ETV+CPC and only 16% used ETV+CPC ≥25% of the time before training. After training in Uganda, the percentage of surgeons who thought ETV+CPC was superior doubled (80% vs. 40%, p=0.2) and the trained surgeons offered ETV+CPC more often as 70% of them now offer ETV+CPC use ≥50% of the time compared to 0% pre-training (p=0.012).

## Conclusion

For the treatment of infant hydrocephalus, there appears to be equipoise regarding the preference of ETV+CPC versus shunt. Training at CURE led to more favorable attitudes and an increased propensity to offer ETV+CPC.

